# Harnessing the Power of Pre-Trained Models for Efficient Semantic Communication of Text and Images †

**DOI:** 10.3390/e27080813

**Published:** 2025-07-29

**Authors:** Emrecan Kutay, Aylin Yener

**Affiliations:** Department of Electrical and Computer Engineering, The Ohio State University, Columbus, OH 43210, USA; yener@ece.osu.edu

**Keywords:** semantic communication, semantic compression, multimodal communication, task-oriented efficiency, 6G

## Abstract

This paper investigates point-to-point multimodal digital semantic communications in a task-oriented setup, where messages are classified at the receiver. We employ a pre-trained transformer model to extract semantic information and propose three methods for generating semantic codewords. First, we propose semantic quantization that uses quantized embeddings of source realizations as a codebook. We investigate the fixed-length coding, considering the source semantic structure and end-to-end semantic distortion. We propose a neural network-based codeword assignment mechanism incorporating codeword transition probabilities to minimize the expected semantic distortion. Second, we present semantic compression that clusters embeddings, exploiting the inherent semantic redundancies to reduce the codebook size, i.e., further compression. Third, we introduce a semantic vector-quantized autoencoder (VQ-AE) that learns a codebook through training. In all cases, we follow this semantic source code with a standard channel code to transmit over the wireless channel. In addition to classification accuracy, we assess pre-communication overhead via a novel metric we term system time efficiency. Extensive experiments demonstrate that our proposed semantic source-coding approaches provide comparable accuracy and better system time efficiency compared to their learning-based counterparts.

## 1. Introduction

6G is envisioned as AI-native, seamlessly integrating human–machine and machine-to-machine communications to support emerging applications such as digital twins, fully autonomous vehicles, and smart environments, all of which are expected to generate and exchange unprecedented data volumes [1,2]. Conventional communication systems treat information exchange as the precise reconstruction of digital sequences at endpoints, without regard to their semantic content [3] and thus rely exclusively on syntactic error metrics such as bit error rate (BER) and symbol error rate (SER). While this semantic-agnostic framework simplifies design and enables modular optimization, it overlooks the meaning of transmitted data, which can be leveraged for improving efficiency. As 6G applications impose ever-more stringent demands for reliability and latency, there is a growing need to incorporate semantic awareness into communication systems to optimize resource utilization and better serve these requirements.

Semantic communication [4] moves in this direction by shifting the focus from transmitting complete sequences to conveying intended meanings. Paying attention to what messages mean enables the transmission of relevant information. Such efforts can potentially enable more efficient use of spectrum resources in future generations of connectivity.

Efforts towards semantics go back as early as shortly after the seminal work in [3], but have achieved limited adoption in communications design [5,6,7,8]. More recently, a decade ago, reference [9] has introduced the concept of *semantic distortion* as an error metric, addressing the transmission of words over noisy channels by optimizing codeword assignments to minimize semantic distortion (loss). The same reference points to the importance of *context* availability at the receiver, which can further improve resource efficiency. Reference [10] builds upon this latter concept and analyzes a system that consists of a transmitter-receiver pair and an external agent that can provide helpful or deceiving context to the receiver. It is important to note that these studies, while fundamental to the concept, were limited to small systems owing to the computational complexity.

Following the machine learning revolution and its entry into the wireless physical layer design [11], efforts in semantic communications were rejuvenated. These efforts adopted deep learning (DL) architectures, structured around four core components: (i) semantic encoder, (ii) channel encoder, (iii) channel decoder, and (iv) semantic decoder [4]. A semantic encoder can extract semantic information from source, generating low-dimensional embeddings using DL models, such as text or vision transformers [12], transmitted over the channel. At the receiver, the semantic decoder processes the reconstructed embeddings to perform tasks from reconstruction to classification, or translation [13,14]. This architecture adopts an autoencoder-based design with end-to-end training with task-specific loss functions [15,16,17,18].

This approach, though a clear paradigm shift, is not without its challenges. Notably, the training overhead can be significant, due to large model size, which requires substantial data [19]. In addition, solutions are often case-specific, which necessitates retraining often in dynamic environments. This creates a training overhead in system design that needs to be addressed. In our preliminary conference papers [20,21], we have demonstrated that utilizing pre-trained embedding models can overcome this limitation.

In this paper, building on these initial ideas, we provide a comprehensive framework for harnessing the power of pre-trained models to digitally communicate semantics extracted from text and images. As an example of this framework, we focus on a task-oriented scenario where the receiver is tasked with classification of said text and images. We concatenate these resulting source codewords by a standard channel coder, this is performed to demonstrate that a backward-compatible, modular, i.e., source-channel separation-based approach [3], is sufficient to obtain significant gains. Our contributions are as follows:

- We propose three semantic codebook generation methods: (i) semantic quantization, which forms a fixed codebook from source realizations; (ii) semantic compression, which builds on quantization by exploiting semantic redundancy; and (iii) semantic vector-quantized autoencoder (VQ-AE), which trains a codebook.
- For semantic quantization and compression, we design a neural network-based source code that minimizes end-to-end semantic distortion.
- We introduce a novel performance metric called *system time efficiency* to account for pre-communication overhead in machine-learning aided wireless communication systems. It quantifies task completion given a time budget by jointly accounting for training and transmission phases, enabling fair comparison and penalizing models with excessive training overhead. Simulations on multiple datasets demonstrate that semantic quantization and compression improve time efficiency over wireless channels, while reducing training cost and enhancing resilience under data scarcity compared to the learning-based semantic VQ-AE.

The rest of the paper is organized as follows. Preliminaries are provided in Section 2, followed by the system model in Section 3. Proposed approaches are presented in Section 4 with numerical results pointing to significant savings against baselines in Section 5. Section 6 concludes the paper.

*Notation:* Lowercase letters, *x*, denote scalars, italic uppercase letters, *X*, denote random variables (RVs), bold lowercase letters, q, denote vectors, and bold uppercase letters, Q, denote matrices. For matrix Q, Q[i] denotes the $i^{th}$ row vector, Q[i,j] denotes the element at $i^{th}$ row $j^{th}$ column, and $Q^{T}$ denotes the transpose. ${∥\cdot ∥}_{2}$ and ${∥\cdot ∥}_{F}$ are the Euclidean and Frobenius norm, respectively. $I_{t\times t}$ denotes identity matrix of rank *t*. ${\left\{0,1\right\}}^{a\times b}$ denotes binary matrix of dimensions a×b. ·modt denotes the modulo operation with respect to *t*. ⌈·⌉ is the ceiling. ${\left(\cdot \right)}_{+}$ denotes the max(·,0). $d_{H}\left(\cdot ,\cdot \right)$ is the Hamming distance. $\log_{2}\left(\cdot \right)$ denotes logarithm with base 2. R and C denotes the real and complex sets. U(a,b) denotes uniform RV in range (a,b). $CN(\mu ,\sigma^{2})$ denotes complex Gaussian RV with mean μ and variance $\sigma^{2}$.

## 2. Preliminaries

In this section, we provide preliminaries of methods we adopt from large language models and unsupervised learning, as these may be less familiar to communications researchers.

Representing text semantics as learned real-valued vectors, i.e., neural embeddings, goes back to *word embeddings*, which capture relationships between words by placing semantically similar words close together in an embedding space [22]. More recently, BERT, one of the first large language models (LLMs), uses the attention mechanism to generate context-aware token embeddings [23], and Sentence-BERT (SBERT) extends this notion to the sentence level by mapping semantically similar sentences to nearby vectors and dissimilar ones to distant vectors [24]. Our preliminary work in references [20,21] utilizes SBERT for semantic text communications.

In this work, our adopted pre-trained model is a recently developed multimodal one. OpenAI CLIP employs transformer-based encoders for both images and text. It uses contrastive learning to jointly train these encoders by predicting the correct (image, text) pairings in a batch. The training objective involves a symmetric loss that equally weights cross-entropies of text-to-image and image-to-text predictions. This ensures consistent embedding properties across both modalities, enabling the unified representation of text and images [25].

Pre-trained embeddings effectively capture semantic information and have demonstrated strong performance across various downstream tasks [26,27]. They serve as compact feature representations, allowing new tasks to be addressed by optimizing only the task-specific block, *without retraining the entire model*. In this work, we use these embeddings to encapsulate message semantics and optimize a classification block for CLIP embeddings.

In unsupervised learning, affinity propagation (AP) is a clustering algorithm that selects centroids from the actual data points, termed “exemplars”. The main objective is to assign each sample to an exemplar by optimizing the defined similarity metric. For minimizing the Euclidean distance, the similarity $sim(o_{i},o_{j})$ between points $o_{i}$ and $o_{j}$ is expressed as follows [28].

(1) $$sim\left(o_{i},o_{j}\right)=-{∥o_{i}-o_{j}∥}_{2}^{2}.$$

The algorithm employs message-passing by exchanging “responsibility” and “availability” messages between data points. The “responsibility” message, r(i,k), is sent from each sample *i* to candidate exemplar *k*, indicating the suitability of *k* as an exemplar for *i*. Correspondingly, the “availability” message, a(i,k), is sent from the candidate exemplar *k* to sample *i*, reflecting the level of suitability for *i* to select *k* as its exemplar. The equations for these messages are as follows [28].

(2) $$r\left(i,k\right)\leftarrow sim\left(i,k\right)-\max_{k^{\prime }s.t.k^{\prime }\neq k}\{a\left(i,k^{\prime }\right)+sim\left(i,k^{\prime }\right)\}.$$

(3) $$a\left(i,k\right)\leftarrow \min\{0,r\left(k,k\right)+\sum_{i^{\prime }s.t.i^{\prime }\notin \left\{i,k\right\}}\max\left\{0,r\left(i^{\prime },k\right)\right\}\},$$

where for a(k,k), the update reduces to $\sum_{i^{\prime }s.t.i^{\prime }\neq k}\max\left\{0,r\left(i^{\prime },k\right)\right\}$. A key advantage of AP is that it does not require the number of clusters to be specified beforehand. Instead, it determines the appropriate number dynamically based on the similarities and message-passing. The number of clusters can be adjusted by changing the preference; setting it to the median of the input similarities typically yields a moderate number of clusters [28].

## 3. System Model

We consider a point-to-point task-oriented communication scenario. The message *s* at the source may be text, image, or a combination of both. The receiver is tasked to predict a class label, denoted by $\hat{c}$. The goal thus is to transmit the essential semantic information needed for accurate classification. Inspired by the use of pre-trained neural models in inverse problems without additional training [29,30], we adopt the OpenAI CLIP transformer as a semantic encoder. This enables us to extract the semantic information of the message as embeddings, denoted by $q\in R^{m}$, without any further training. The (digitized) CLIP embedding thus becomes the message to be sent to the receiver for classification.

We use semantic distortion [9], denoted as δ(·,·), to quantify the loss in meaning. For a message *s* with embedding q, the semantic distortion is

(4) $$\delta \left(q,\tilde{q}\right)={∥q-\tilde{q}∥}_{2},$$

where $\tilde{q}$ is the receiver’s estimate.

Consider a dataset, denoted as $Q_{pre}^{n}$, consisting of embeddings of randomly selected messages. This dataset serves as the initial codebook with *n* codewords of length *m* each, where *n* represents the number of embeddings and *m* is the embedding dimension. We will investigate several approaches to utilize $Q_{pre}^{n}$ for semantic quantization, semantic compression, and semantic VQ AE proposed in Section 4.1, Section 4.2 and Section 4.3.

For a channel input $x\in C^{b}$, the channel output $y\in C^{b}$ is as follows.

(5) y=hx+z,

where $z∼CN(0,\sigma_{z}^{2}I_{b\times b})$ denotes the additive noise, and h∈C represents the fading coefficient.

We employ a neural network (NN) with fully connected layers for the classification block at the receiver. This block takes the reconstructed CLIP embeddings as input for classification.

## 4. Proposed Methods

In this section, we propose three semantic representations, each using the OpenAI CLIP to generate embeddings from messages and incorporating the available dataset $Q_{pre}^{n}$.

### 4.1. Semantic Quantization

We propose first using $Q_{pre}^{n}$ as a shared codebook by arguing that the semantic structure of the source can be inferred from its realizations. This approach encodes messages with actual content (source samples), enabling the interpretability of codeword meanings and their alignment with human understanding, as discussed in Section 5.2. Through semantic quantization, we assign message embeddings to codebook indices that minimize the semantic distortion. In particular, we obtain the codebook index for message embedding q by the semantic index assignment operation, denoted by $\Pi_{Q_{pre}^{n}}\left(\cdot \right)$, as follows.

(6) $$\Pi_{Q_{pre}^{n}}\left(q\right)=\underset{i^{\prime }s.t.0\leq i^{\prime }<n}{\mathrm{argmin}}\delta (q,Q_{pre}^{n}\left[i^{\prime }\right]).$$

**Figure 1:**
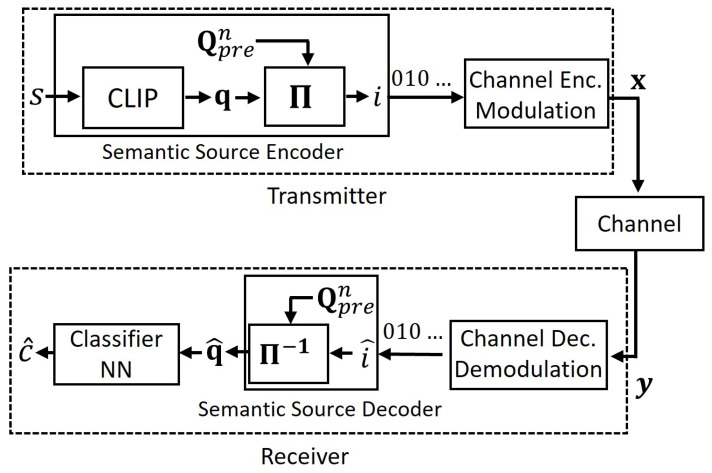
The proposed semantic quantization method.

The (source) codeword is then mapped using a standard channel code, that is we have a backward compatible design with existing digital communications systems: a (semantic) source coder followed by a channel coder. At the receiver, the index is reconstructed as $\hat{i}$ and the associated embedding $\hat{q}=Q_{pre}^{n}\left[\hat{i}\right]$ is passed to the classifier to obtain the prediction $\hat{c}$. We illustrate this process in Figure 1.

We explore the fixed-length coding scheme inspired by an early approach that incorporates channel effects into the semantic distortion objective and exploits semantic relationships among source symbols to improve noise robustness [9]. In particular, we investigate the distortion in Equation (4) by incorporating the codeword transition probabilities over the noisy channel. Denote the codewords as $W\in {\left\{0,1\right\}}^{n\times l_{cw}}$, where $j^{th}$ row contains the codeword assigned to index *j*, with a fixed length of $l_{cw}=\left\lceil \log_{2}n\right\rceil$. The expected semantic distortion Δ(·) is as follows [9]:

(7) $$\Delta \left(W\right)=\sum_{i=0}^{n-1}p\left(i\right)(\sum_{j=0}^{n-1}p(\hat{W}\left[j\right]|W\left[i\right])\delta (Q_{pre}^{n}\left[i\right],Q_{pre}^{n}\left[j\right])).$$

In Equation (7), the distortion and index probability terms are fixed. We focus on minimizing the objective by choosing the codewords, which determine the channel transition probabilities $p(\hat{W}\left[j\right]|W\left[i\right])$. This makes the problem similar to minimizing a linear function over a probability simplex; it is easy to conclude that we need codewords such that higher transition probabilities correspond to lower semantic distortion. To achieve this goal, we design the codewords by linking transition probabilities to Hamming distances, where smaller Hamming distances indicate higher transition probabilities. This serves as a model-based insight guiding our design. Therefore, the objective is to assign codewords such that the Hamming distance $d_{H}\left(W\left[i\right],W\left[j\right]\right)$ reflects the Euclidean distance between their embeddings, ensuring that closer indices have codewords with smaller Hamming distances.

Building on this model-based intuition, we propose the NN-based codeword assignment mechanism to minimize the expected distortion in Equation (7). Specifically, we consider an NN, which takes $Q_{pre}^{n}$ as input and aligns codeword Hamming distances with a tanh(·) output activation, as follows.

(8) $$\tilde{W}=g\left(Q_{pre}^{n};\theta \right),$$

where θ is the learnable parameter vector, and $\tilde{W}\in R^{n\times l_{cw}}$ represents the NN soft outputs, which are binarized afterward to form the codewords. To align these assignments with the semantic relationships between indices, we design the loss function $L_{\mathrm{cw}}$ as a convex combination of multiple components. In particular, we optimize the following loss function through mini-batches of size $n_{b}$.

(9) $$\begin{array}{cc} L_{\mathrm{cw}} & =\alpha_{1}L_{\mathrm{Triplet}}+\alpha_{2}L_{\mathrm{Diversity}}+\alpha_{3}L_{\mathrm{Orthogonality}}\\ \; & =\frac{\alpha_{1}}{n_{b}}\sum_{j=0}^{n_{b}-1}(∥{\tilde{W}}_{b}\left[j\right]-{\tilde{W}}_{b}\left[p_{j}\right]{∥}_{2}-∥{\tilde{W}}_{b}\left[j\right]-{\tilde{W}}_{b}\left[n_{j}\right]{{∥}_{2}+\epsilon )}_{+}\\ \; & +\frac{\alpha_{2}}{n_{b}^{2}}\sum_{i,j=0}^{n_{b}-1}{\left(\sqrt{l_{cw}}-D_{{\tilde{W}}_{b}}\left[i,j\right]\right)}_{+} \end{array}$$

(10) $$\begin{array}{cc} \; & +\frac{\alpha_{3}}{n_{b}^{2}}{∥{\tilde{W}}_{b}{\tilde{W}}_{b}^{T}-I_{n_{b}\times n_{b}}∥}_{F}^{2}, \end{array}$$

where ${\tilde{W}}_{b}$ is the batch soft output, $p_{j}=p_{s}\left[j\right]$ and $n_{j}=n_{s}\left[j\right]$ are the positive and negative sample indices of sample *j* as in Algorithm 1, ϵ is the margin, $D_{{\tilde{W}}_{b}}$ is the pairwise distance matrix in Algorithm 2. We design codewords by adjusting soft output distances so that outputs with close Euclidean distances are binarized to codewords with small Hamming distances. We use triplet loss to align soft outputs with positions of embeddings in $Q_{pre}^{n}$ associated with indices. Specifically, we generate triplets such that positive and negative samples correspond to closer and farther embeddings in a batch, as outlined in Algorithm 1. Instead of generating fixed triplets, we repeatedly generate new ones for each batch, helping the model to align better by adapting to dynamic pairs. As the triplet loss can lead to duplicate assignments, we introduce regularization terms to the loss function to penalize the model for producing outputs that cluster too densely. We provide the training procedure in Algorithm 2.
**Algorithm 1** Triplet Generation for Triplet Loss in Each Batch
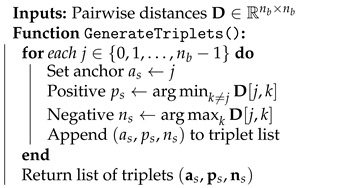



**Algorithm 2** Training Codeword Assignment Model

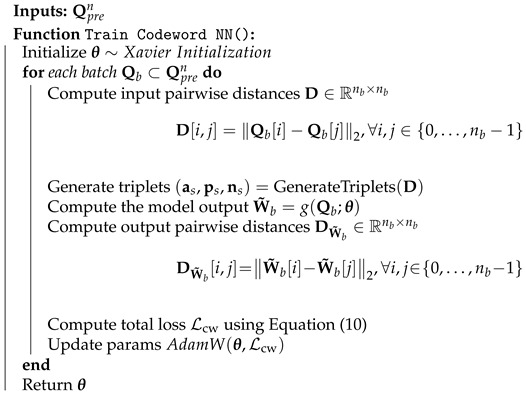




After training, we pass $Q_{pre}^{n}$ through the model, as in Equation (8), to obtain the soft outputs. We then binarize them based on their sign, resulting in ${\tilde{W}}_{bin}$. Although we have designed the loss to encourage distinct assignments, this does not directly ensure unique decodability. To address this, we map ${\tilde{W}}_{bin}$ to distinct codewords of the same length by minimizing the Hamming distance, formulating the problem as follows.

(11) $$W_{opt}=\underset{W}{arg min}\sum_{i=0}^{n-1}d_{H}\left(W\left[i\right],{\tilde{W}}_{bin}\left[i\right]\right)\;s.t.\;\begin{array}{c} W[i]\neq W[j]\\ \forall i\neq j \end{array}.$$

The problem in Equation (11) is a linear sum assignment problem, which involves minimizing the total assignment cost over one-to-one mappings between agents and tasks [31]. We define the cost by the pairwise Hamming distances between the ${\tilde{W}}_{bin}$ and unique codewords to be assigned $W_{opt}$. In particular, we construct a cost matrix $D^{H}$ as given in Algorithm 3. However, when *n* is not a power of 2, we add dummy rows with a cost greater than the maximum of non-dummy rows. This ensures the use of all distinct codewords of length $l_{cw}$. Utilizing this cost matrix, we solve the problem using a modified Jonker-Volgenant algorithm [32], an efficient variant of the Hungarian algorithm that improves time complexity. We summarize the complete process in Algorithm 3.

### 4.2. Semantic Compression

In Section 4.1, increasing *n* improves resolution in $R^{m}$ for quantization, but leads to a larger codebook. Semantics contain inherent redundancies, where the meaning is the same despite variations in sentence structures, or even words [33]. To reduce the codebook size while maintaining resolution, we propose semantic compression, which clusters similar embeddings in $Q_{pre}^{n}$, exploiting these semantic redundancies.

**Figure 2:**
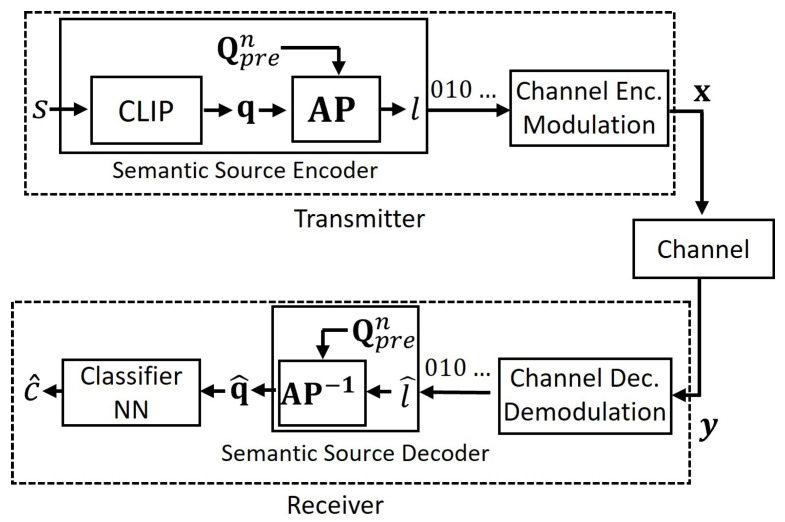
The proposed semantic compression method.

In particular, we use the affinity propagation (AP) algorithm for clustering explained in Section 2 which identifies the optimal number of clusters. We assign message embeddings to the resulting clusters by minimizing semantic distortion, as follows.

(12) $${\mathrm{AP}}_{Q_{pre}^{n}}\left(q\right)=\underset{l^{\prime }s.t.0\leq l^{\prime }<\tilde{n}}{\mathrm{argmin}}\delta (q,Q_{pre}^{\tilde{n}}\left[l^{\prime }\right]),$$

where $Q_{pre}^{\tilde{n}}$ contains $\tilde{n}$ centroids with $\tilde{n}\ll n$. The codeword corresponding to the assigned cluster label, *l*, is transmitted using the same transmission scheme in Section 4.1. At the receiver, the reconstructed cluster label, $\hat{l}$, is used to retrieve the associated embedding $\hat{q}=Q_{pre}^{\tilde{n}}\left[\hat{l}\right]$, which is then fed into the classifier to obtain the prediction $\hat{c}$, as illustrated in Figure 2.
**Algorithm 3** Codeword Assignment for Codebook Indices
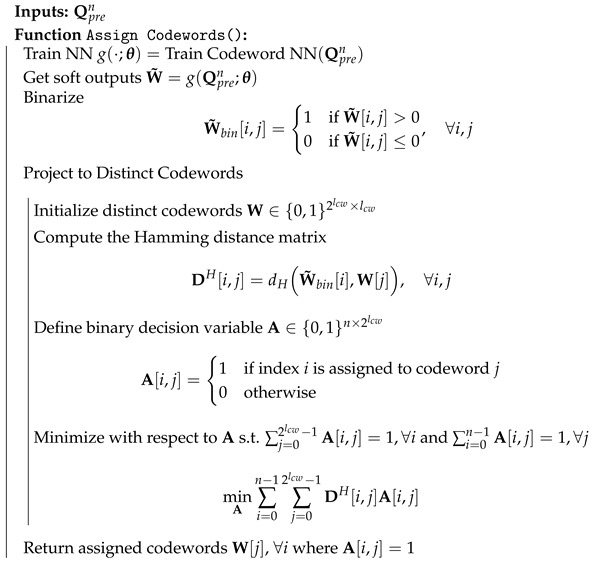


We employ Algorithms 1–3 for a fixed-length coding of cluster labels similar to the Section 4.1. However, instead of utilizing $Q_{pre}^{n}$ to design codewords of length $l_{cw}=\left\lceil \log_{2}n\right\rceil$, here we use clustered version $Q_{pre}^{\tilde{n}}$ to design codewords of length ${l^{\prime }}_{cw}=\left\lceil \log_{2}\tilde{n}\right\rceil$.

### 4.3. Semantic Vector-Quantized Autoencoder

In this section, we focus on learning-based semantic communication by using $Q_{pre}^{n}$ as training data instead of directly employing it as a codebook. Drawing inspiration from the vector-quantized variational autoencoder (VQ VAE) [34], we propose the semantic vector-quantized autoencoder (VQ AE) to generate learned discrete representations for message embeddings. The VQ AE consists of an encoder, a decoder, and a codebook $E\in R^{d\times k}$, which contains *d* latent space points for quantizing the encoder’s output vectors. In particular, the encoder maps the message embedding to a latent vector, denoted as $v\in R^{k}$, expressed as follows.

(13) $$v=v_{enc}\left(q;\gamma_{enc}\right),$$

where $\gamma_{enc}$ denotes the learnable parameter vector of the encoder. In the latent space, the vector v is then quantized by assigning it to one of the elements in the codebook E, as follows.

(14) $$\mathrm{CD}\left(v\right)=\underset{p^{\prime }s.t.0\leq p^{\prime }<d}{\mathrm{argmin}}{∥v-E\left[p^{\prime }\right]∥}_{2}.$$

**Figure 3:**
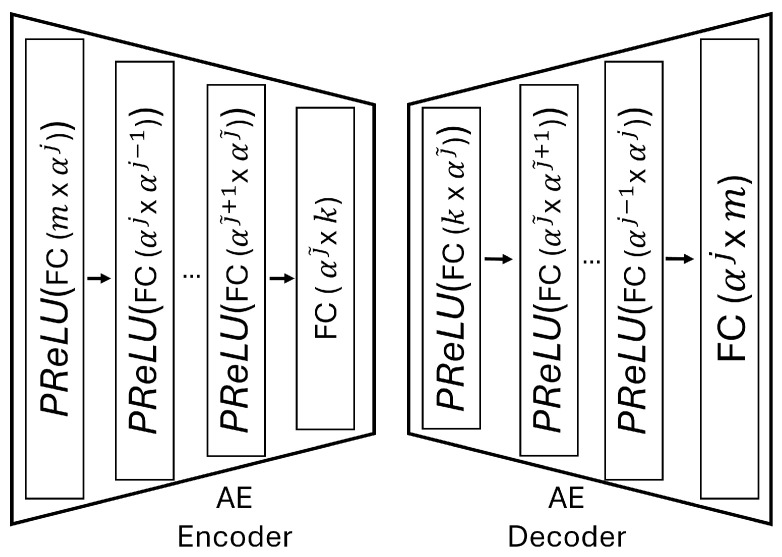
Structure of the AE encoder–AE decoder in semantic VQ AE.

Equation (14) assigns the codebook element for v, denoted as e=E[p], where *p* is the index of the selected element. We then pass e through the decoder to reconstruct the embedding, $\hat{q}$, as 

(15) $$\hat{q}=v_{dec}\left(e;\gamma_{dec}\right),$$

where $\gamma_{dec}$ is the learnable parameter vector of the decoder. In this architecture, the encoder and decoder constitute an autoencoder, both employing a neural network with fully connected (FC) layers and a parameter α. In the encoder, the initial FC layer maps embeddings to $\alpha^{j}$-dimensional space, then iteratively scales down dimensions by $\alpha^{-1}$ until reaching $\alpha^{\tilde{j}}$. Here, *j* and $\tilde{j}$ are chosen such that *j* represents the largest number where $\alpha^{j}<m$, and $\tilde{j}$ represents the smallest number where $k<\alpha^{\tilde{j}}$. The final FC layer maps directly to $R^{k}$, i.e., to the latent space. Conversely, the decoder mirrors the encoder’s architecture, progressively increasing dimensions to reconstruct the embeddings from the codebook elements. The parameter α>1 determines the model’s depth, with smaller values yielding more layers. We illustrate this structure in Figure 3, employing PReLU(·) as the activation function. We optimize the encoder, decoder, and codebook using the VQ-VAE training algorithm, employing the following loss function [34].

(16) $$L_{\mathrm{AE}}=||q-\hat{q}{\left|\right|}_{2}^{2}+||sg\left(v\right)-{e||}_{2}^{2}+\beta ||v-{sg\left(e\right)||}_{2}^{2},$$

where β represents the commitment parameter, and sg(·) denotes the stop gradient operator, preventing parameter updates by ensuring zero partial derivatives. The first term is the reconstruction loss for accurate embedding reconstruction at the decoder. The codebook is learned through the second term, minimizing the distance between e and v. The final term, i.e., commitment loss, penalizes the encoder for deviating from the codebook elements [34].

In Section 4.1 and Section 4.2, we have designed an NN-based codeword assignment mechanism. Here, we utilize adversarial training [35]. This approach provides latent space points that are resilient to channel effects. Specifically, we simulate channel conditions by perturbing selected index *p* to $\tilde{p}$ and passing the perturbed latent vector, $\tilde{e}=E\left[\tilde{p}\right]$, through the decoder. Unlike in previous sections, we note that the channel effects here directly modify the discrete representations in the latent space. We provide the details of the perturbation in Algorithm 4 and the training algorithm of the semantic VQ AE in Algorithm 5.
**Algorithm 4** Index Perturbation for Adversarial Training
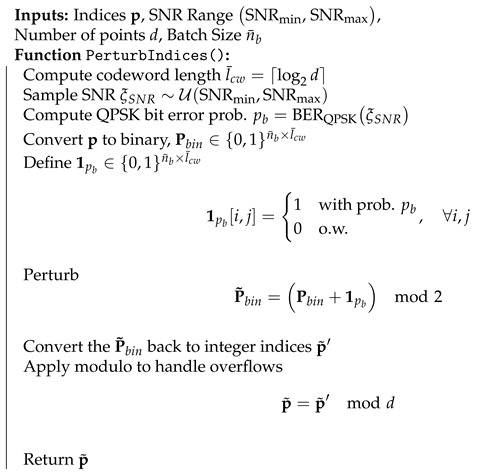


**Figure 4:**
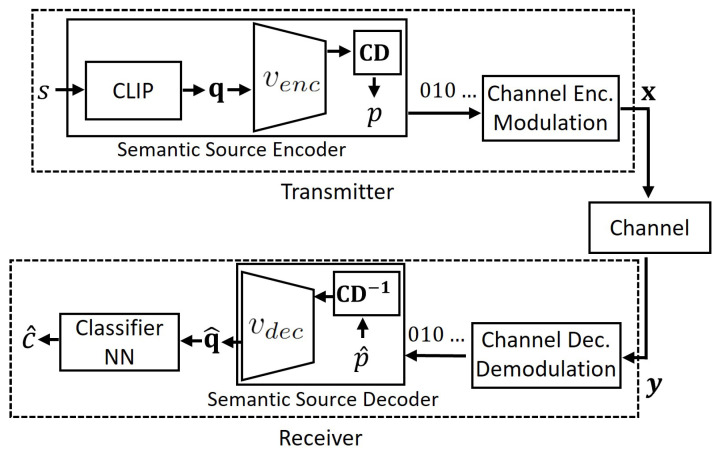
The proposed semantic VQ AE method.

After training the model on the loss in Equation (16), the learned codebook and encoder–decoder components are shared between the transmitter and receiver. We then assign message embeddings to the learned codebook elements by using Equations (13) and (14). The resulting index *p* is converted to binary and transmitted using the same transmission scheme in Section 4.1 and Section 4.2. At the receiver, reconstructed index $\hat{p}$ retrieves $\hat{e}=E\left[\hat{p}\right]$ which is used to reconstruct $\hat{q}$ through Equation (15) and then obtain the prediction $\hat{c}$. We illustrate this process in Figure 4.
**Algorithm 5** Training Semantic VQ AE Model
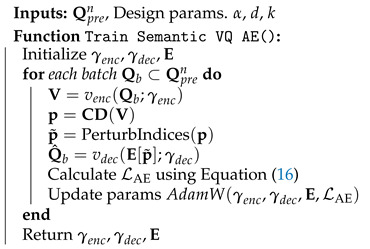


### 4.4. Performance Metrics

The task in this paper is classification. Naturally, classification accuracy is the performance metric against which to measure the proposed methods. In addition, we introduce a novel performance metric that enables comparing pure deep learning-based approaches and the approaches we propose utilizing pre-trained models. *System time efficiency*, denoted by $\eta_{T}$, quantifies the number of correct task executions in a specified time frame, including the training phase, hence providing a measure for the pre-communication overhead of various machine learning methods. Specifically, for a method that achieves *u* correct classifications per second following a training phase of $t_{train}$ seconds, we define $\eta_{T}$ for a time budget $\tilde{t}$ as follows.

(17) $$\eta_{T}(\tilde{t})=\left(\tilde{t}-t_{train}\right)u.$$

In Equation (17), the higher $\eta_{T}\left(\cdot \right)$ the better the efficiency. This metric addresses a gap often overlooked in recent works, where the computational cost of training is not explicitly included in comparisons with conventional systems.

## 5. Results

In this section, we present the numerical results for source compression and transmission over wireless channels.

### 5.1. Datasets and Simulation Settings

We evaluate the proposed methods using four classification datasets: (i) AG’s News [36], which contains news articles from 4 categories; (ii) DBPedia 14 [36], consisting of text samples from 14 ontology classes; (iii) CIFAR 10 [37], including 32×32 RGB images of 10 object categories; and (iv) STL 10 [38], with 96×96 RGB images from 10 object categories. We present details of these datasets in Table 1. For consistency, we test on 2000 balanced samples with respect to class labels for each dataset. We employ the clip-ViT-B-32 as the CLIP model to obtain message embeddings.

The classifier NN involves three layers with 128, 32, and $n_{class}$ hidden units, where $n_{class}$ corresponds to the number of classes in the dataset. We train the NN on the receiver’s dataset, which consists of message embeddings, $q_{train}$, and their corresponding labels, $c_{train}$.

In the semantic compression method, we use the negative Euclidean distance, defined in Equation (1), as the similarity metric to minimize semantic distortion in Equation (4). To determine the optimal number of clusters, we use the median of sample similarities as the preference, as discussed in Section 2. The codeword neural network (NN) has a simple structure consisting of three fully connected layers with 256, 64, and $l_{cw}$ units (${l^{\prime }}_{cw}$ for semantic compression). We use PReLU(·) activation in the hidden layers and tanh(·) at the output. For semantic VQ AE, we set *d* to match the number of clusters determined by the AP algorithm, as given in Table 3. This ensures a fair comparison among methods. We determine the optimal values through grid search k∈{16,64,256,512} and α∈{2,3,4,5}, primarily considering model performance, while also accounting for model size and computational complexity. We set the commitment parameter in Equation (16) to β=0.25. To optimize both classification accuracy and expedite the training process, we increase the learning rate and adjust the number of epochs for proper optimization in fewer steps.

Table 2 lists all the parameters used in simulations. We construct $Q_{pre}^{n}$ by selecting *n* samples from the shuffled training data. The remaining training samples constitute the receiver dataset, denoted as $Q_{train}$ and $c_{train}$. For the classification block training, we split $Q_{train}$ and $c_{train}$ into training and validation sets with ratios of 0.85 and 0.15, respectively. We use the model with the minimum validation loss achieved across the training epochs. We simulate the multimodal scenario by combining 2500 samples each from the AG’s News and STL 10 datasets, forming $Q_{pre}^{n}$ with n=5000. We assume the receiver uses separate classifiers for images and text, trained with the same parameters as those used for the original datasets, as given in Table 2. Training procedures of the codeword NN and semantic VQ AE have the same parameters in the AG’s News dataset, except for $\gamma_{scheduler}=0.95$ and k=16 for semantic VQ AE. We simulate the multimodal case using 2000 test samples from each dataset, resulting in 4000 in total, which are also balanced with respect to class labels.

We implement conventional source-coding baselines to investigate the efficacy of the proposed semantic methods. We employ Huffman coding for text compression and JPEG2000 for image compression. We adopt block coding for Huffman, where compression symbols consist group of three characters. We derive empirical probabilities for these blocks from the provided text. Regarding the JPEG2000 algorithm, we use the Python Pillow package (version 9.3.0), adhering to its default parameters, except for configuring the quality layers parameter, set to 20. As these algorithms ensure exact reconstruction, we assess their accuracy using the original embeddings of messages, without any form of quantization or compression.

For wireless channel simulations, h=1 for the AWGN channel and $h∼CN(0,\sigma_{h}^{2})$ for Rayleigh fading channels with $\sigma_{h}^{2}=1$. We employ an average power constraint of 1 for all transmission methods. Consequently, the channel signal-to-noise ratio (SNR) becomes $-10\log_{10}\left(\sigma_{z}^{2}\right)$ dB. We adjust the noise variance, $\sigma_{z}^{2}$, to emulate average channel SNR values. We employ transmit power control (channel inversion) to mitigate the effects of fading.

**Table 3:** Number of bits required to compress test messages and their classification accuracies for fixed-length coding (mean ± 1 SD, 10 runs).

Dataset	Method	Number of Bits	Acc. (%)
AG’s News	Conventional	1,480,599	92.50±0.08
	Sem. Quan.	26,000	88.04±0.65
	Sem. Comp. 501 *	18,000	85.25±0.87
	Sem. VQ AE 501 ^+^	18,000	84.23±0.67
DBPedia 14	Conventional	2,160,054	98.60±0.08
	Sem. Quan.	24,000	90.44±0.35
	Sem. Comp. 252 *	16,000	85.78±0.45
	Sem. VQ AE 252 ^+^	16,000	83.51±0.97
CIFAR 10	Conventional	4,073,328	95.90±0.08
	Sem. Quan.	24,000	89.08±0.56
	Sem. Comp. 147 *	16,000	86.13±0.53
	Sem. VQ AE 147 ^+^	16,000	84.26±0.94
STL 10	Conventional	22188432	98.55±0.09
	Sem. Quan.	22,000	97.95±0.28
	Sem. Comp. 88 *	14,000	97.31±0.18
	Sem. VQ AE 88 ^+^	14,000	95.39±1.09
Multi Modal	Conventional	23,659,964	95.67±0.12
	Sem. Quan.	52,000	92.20±0.34
	Sem. Comp. 208 *	32,000	89.45±0.56
	Sem. VQ AE 208 ^+^	32,000	85.60±1.16

* The number of clusters determined by the AP algorithm. ^+^ The size of E, i.e., the value of *d* in semantic VQ AE.

We conduct simulations on a computer equipped with an Intel Core i9-13950HX CPU at 1.6 GHz and an NVIDIA GeForce RTX 4070 GPU, which is employed to accelerate neural model training. To ensure reliable results, we measure the simulation times while the computer is idle and all background applications are disabled. For each method, we repeat the simulations 10 times and present the mean scores with standard deviations (SD) to quantify the uncertainty. In each run, we use a different seed to initialize the neural network parameters and shuffle the data, resulting in distinct $Q_{pre}^{n}$ across runs.

### 5.2. Source Compression

**Figure 5:**
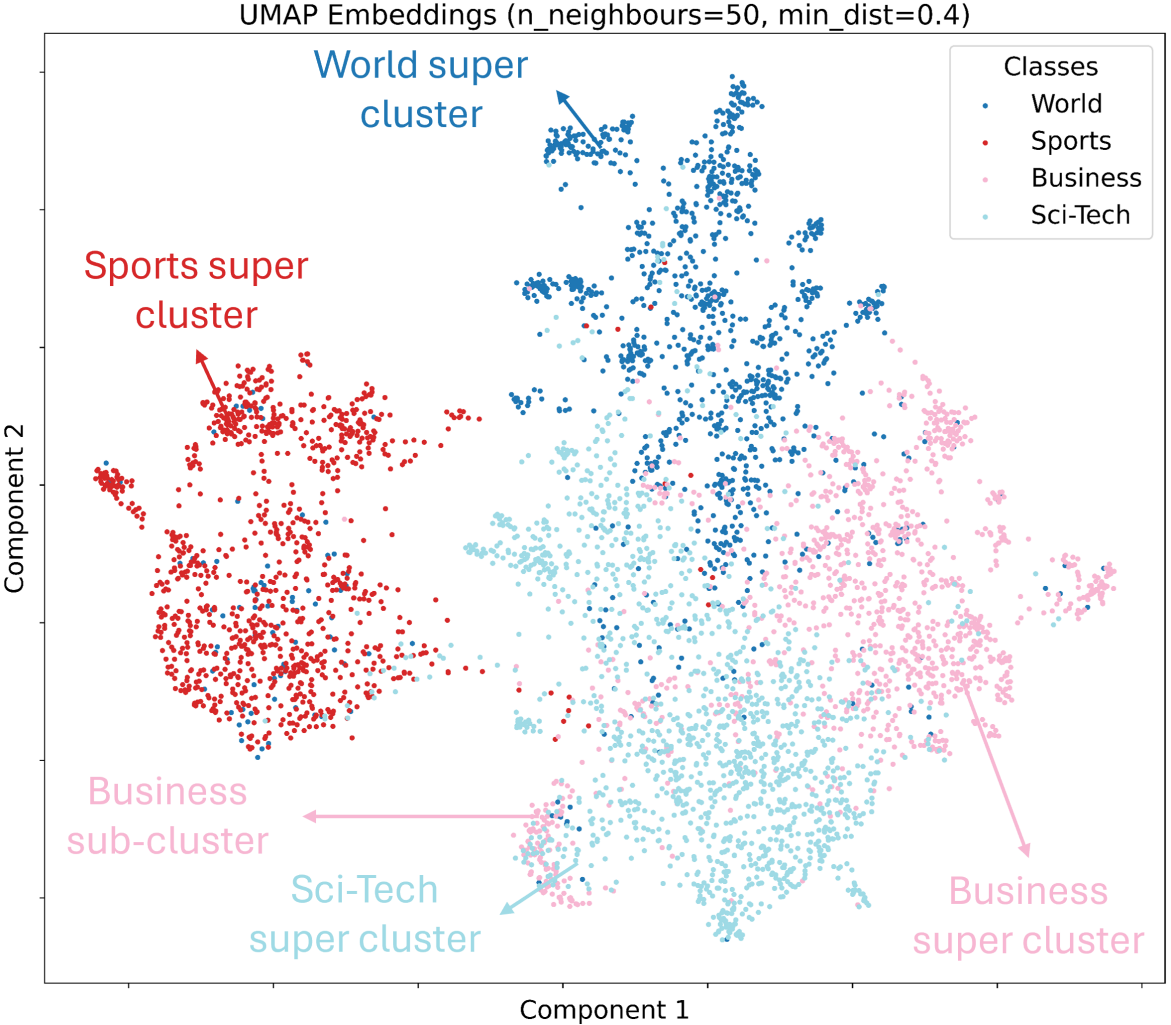
UMAP projection of AG’s News $Q_{pre}^{n}$ embeddings into $R^{2}$.

We start by examining the structure of pre-trained embeddings in $Q_{pre}^{n}$. To visualize, we project the embeddings into $R^{2}$ using uniform manifold approximation and projection (UMAP) [39]. As we have described in Section 3, $Q_{pre}^{n}$ consists of random samples without any prior knowledge. However, just for this visualization, we use the class information of embeddings to illustrate their structure. Figure 5 indicates that embeddings from the same class tend to cluster together. This supports the intuition that class-related semantics can be preserved, even after clustering.

Table 3 presents the source compression results, including the number of bits required to compress test samples and their associated classification accuracies. We observe that the proposed semantic methods improve resource efficiency compared to conventional baselines. Regarding classification accuracies, semantic quantization attains the highest score, likely due to its larger codebook, which offers finer semantic resolution at the cost of higher bit usage. By contrast, semantic compression maintains comparable accuracy while using fewer bits, effectively preserving resolution by removing redundancy. These findings confirm that exploiting semantic redundancy reduces data size without a major accuracy loss. Furthermore, as detailed in Section 5.1, we ensure a fair comparison between semantic compression and semantic VQ AE in terms of resource utilization by having the same codebook size. Under these equal conditions, we observe that semantic compression achieves higher classification accuracies than semantic VQ AE. This suggests that, given the same resource constraints, semantic compression better preserves class-related semantics while utilizing the same amount of resources. These observations are consistent across all datasets, including the custom multimodal dataset. This highlights the feasibility of constructing a unified multimodal codebook consisting of codewords for both image and text messages. Overall, the results demonstrate that semantic quantization and compression methods can generalize across diverse contexts and modalities without requiring separate training for each to obtain learned discrete representations.

The number of clusters determined by the AP algorithm in Table 3 is significantly large. We attribute this to the high dimensionality of the embeddings (m=512) and the existence of small sub-clusters in broader super-clusters. For example, Figure 5 demonstrates a small group from the “Business” class embedded in the “Sci-Tech” class, leading to an additional cluster.

In Table 4, we select a random sample from the AG’s News test set denoted as *s*, and retrieve the messages corresponding to the assigned index and cluster label denoted as $s_{i}$ and $s_{l}$, respectively. A comparison of these messages reveals a strong alignment with human understanding, reinforcing the interpretability discussed in Section 4.1 and Section 4.2. This alignment suggests that the semantics required for the classification task are effectively conveyed while enhancing efficiency, which is the aim of this work.

**Figure 6:**
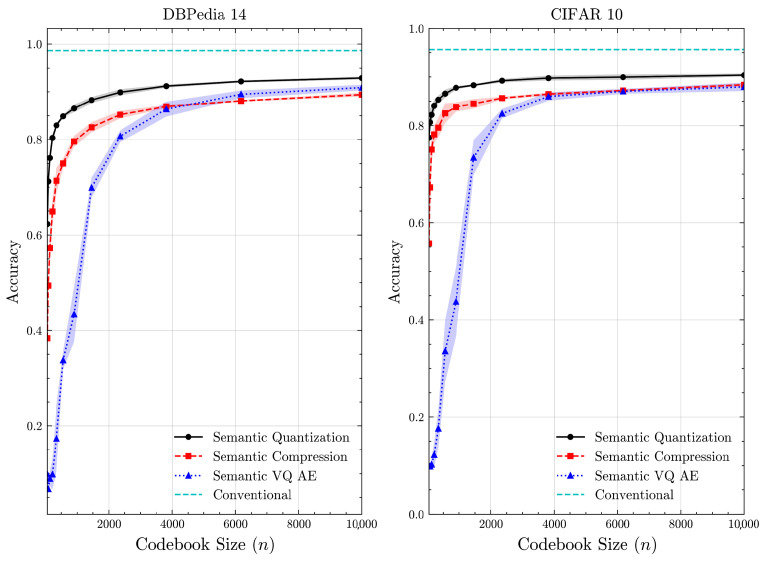
Classification accuracy vs. *n* for DBPedia 14 and CIFAR 10 datasets (mean ± 1 SD, 10 runs).

We examine how the value of *n* impacts performance. While a larger *n* demands more computational resources, *n* needs to be sufficiently large to ensure an adequate number of samples per class for effective codebook construction. Figure 6 provides the accuracy of methods as *n* varies from 50 to 10,000 for the DBPedia 14 and CIFAR 10 datasets. For 3000<n, the accuracy plateaus for all methods, indicating diminishing returns. The proposed semantic quantization and compression methods achieve rapid convergence in accuracy. They perform well even with smaller datasets and demonstrate robustness to varying data sizes. By contrast, semantic VQ AE struggles to achieve high accuracy and converges more slowly. This highlights the data-greedy nature of learning-based approaches, which are sensitive to data size during optimization.

### 5.3. Wireless Channel Simulations

In this section, we evaluate the proposed methods over wireless channels, assuming the exact channel state for transmit power control, i.e., $\hat{h}=h$. We employ Reed–Solomon coding with rate RS(31, 25) and quadrature phase shift keying (QPSK) modulation. We use the accuracy of the conventional approach at infinite SNR as the upper bound, representing lossless transmission. We examine the convergence of the semantic methods to this limit.

We begin by examining the impact of incorporating codeword transition probabilities. To this end, we establish baselines for semantic quantization and compression. In these baselines, the resulting *i* and *l* values are directly encoded into binary format and transmitted with RS(31, 25). For the semantic VQ AE, we train the baseline in a noiseless setup, i.e., without any perturbations to *p*. In this comparison, we have the same number of channel uses for all schemes, as the codeword lengths and the channel coding rates are the same. Thus, any observed improvement in accuracy is attributed to integrating transition probabilities in the system design.

**Figure 7:**
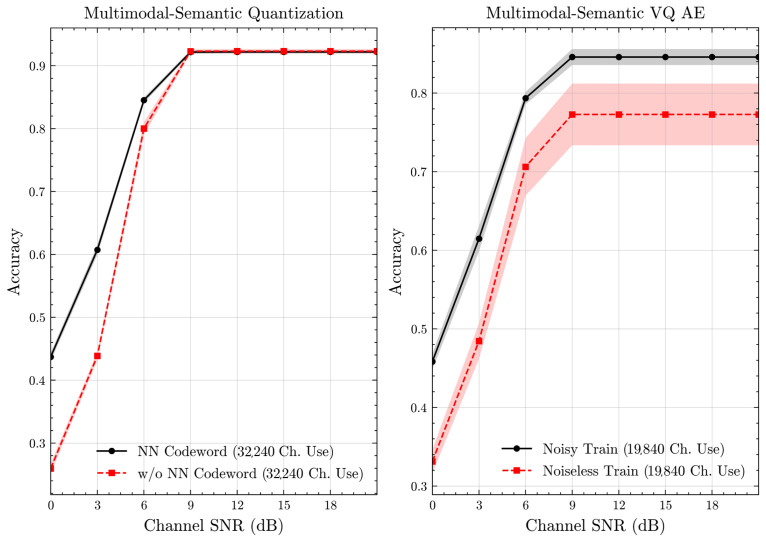
Classification accuracies for the multimodal dataset over AWGN channel (mean ± 1 SD, 10 runs).

**Figure 8:**
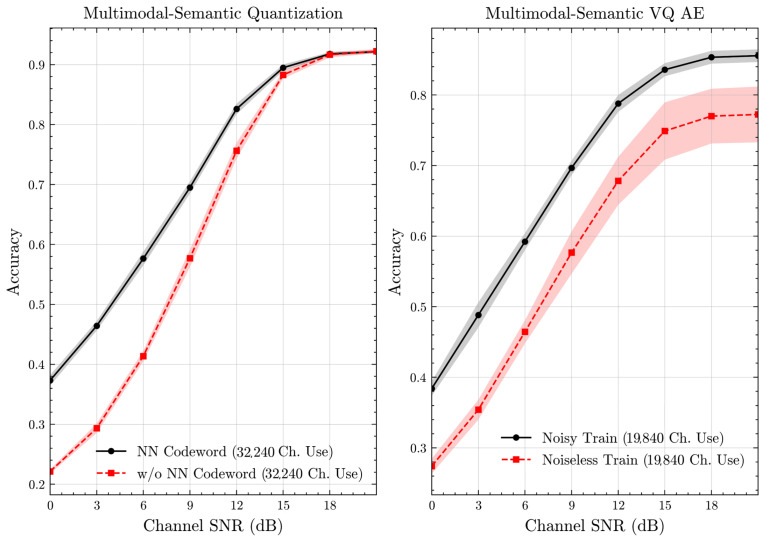
Classification accuracies for the multimodal dataset over Rayleigh channel (mean ± 1 SD, 10 runs).

**Figure 9:**
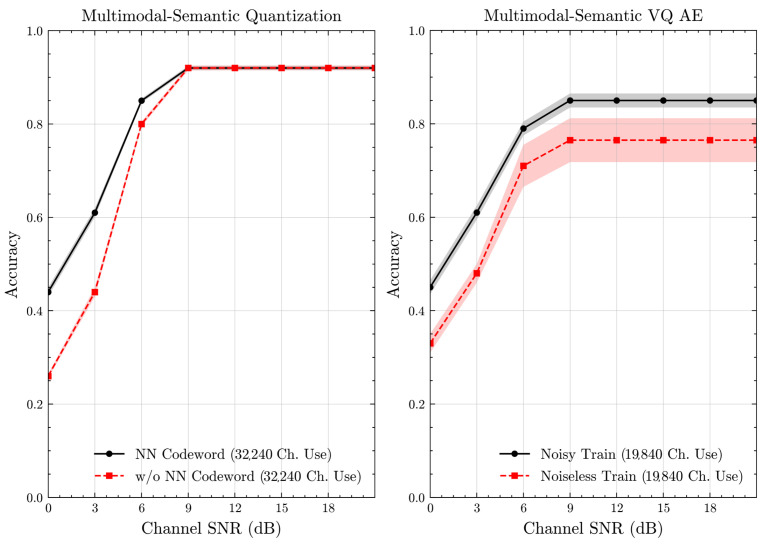
Classification accuracies with full accuracy range for the multimodal dataset over AWGN channel (mean ± 1 SD, 10 runs).

**Figure 10:**
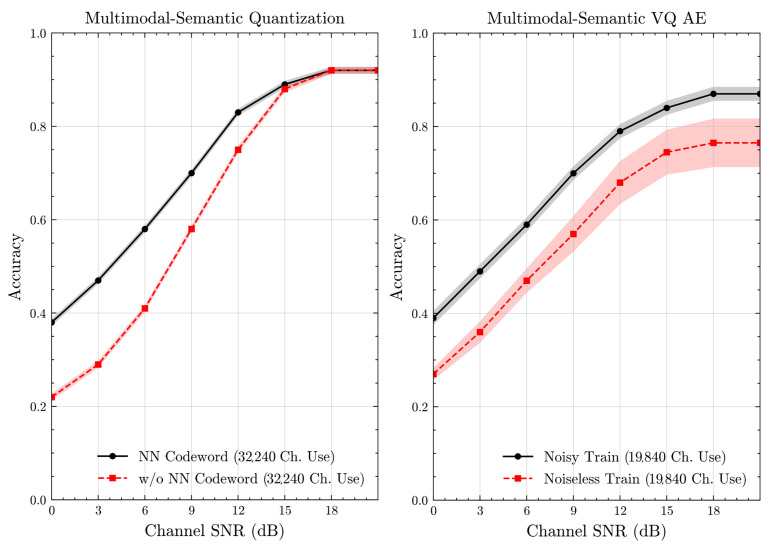
Classification accuracies with full accuracy range for the multimodal dataset over Rayleigh channel (mean ± 1 SD, 10 runs).

Figure 7 and Figure 8 illustrate the accuracies of designed codewords over wireless channels for semantic quantization and noisy training in the semantic VQ AE. Figure 9 and Figure 10 further present these accuracies with the full accuracy range [0,1], as requested by the anonymous reviewer. For the semantic quantization, we observe a significant accuracy improvement in the low SNR region compared to the baseline. This is achieved without adding redundancy and decreasing the rate, making the result promising. The accuracy is the same for high SNR regions, as the proposed codeword assignment mechanism does not interfere with the discrete message representations. For the semantic VQ AE, we observe increased accuracy in low SNR regions, highlighting the benefits of adversarial training. However, a performance gap emerges between noisy and noiseless training in the high SNR region. Unlike semantic quantization, noisy training in semantic VQ AE impacts the discrete representations assigned to codewords. Thus, we interpret this gap as the channel simulation during training acting as a regularizer. Given the small training dataset, the limited number of samples can lead to overfitting. Hence, introducing a regularizer improves performance and ensures more robust discrete representations.

**Figure 11:**
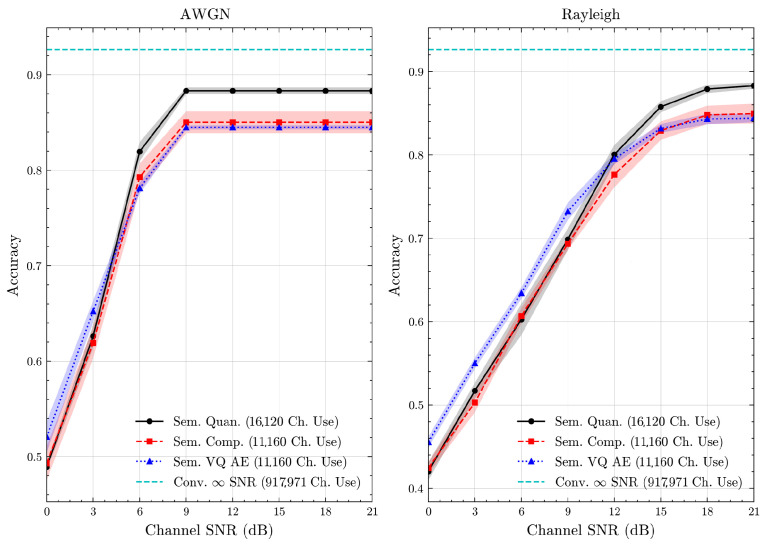
AG’s News dataset classification accuracies over AWGN and Rayleigh channels (mean ± 1 SD, 10 runs).

**Figure 12:**
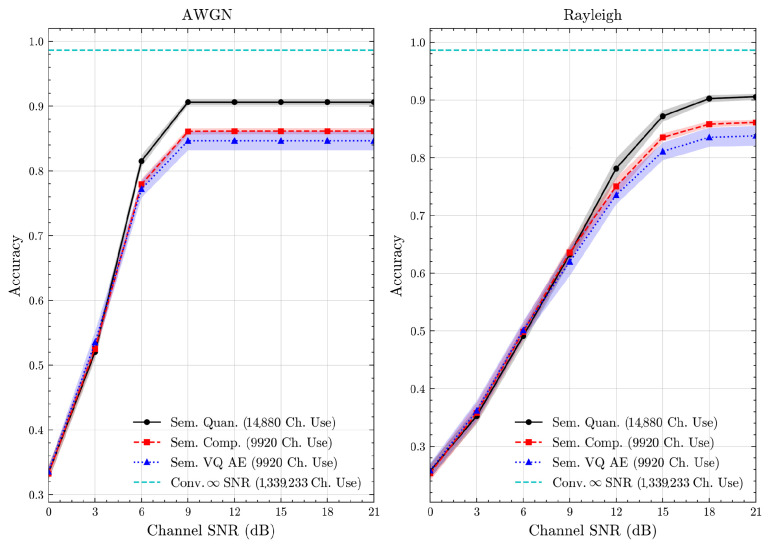
DBPedia 14 dataset classification accuracies over AWGN and Rayleigh channels (mean ± 1 SD, 10 runs).

**Figure 13:**
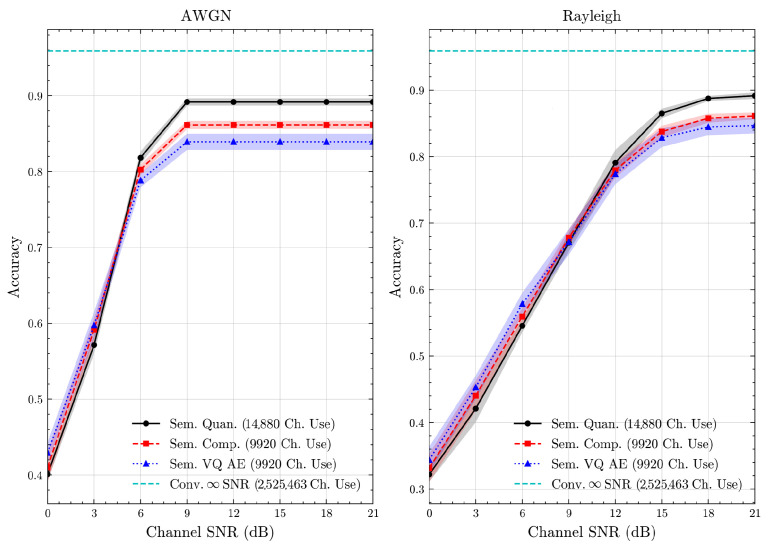
CIFAR 10 dataset classification accuracies over AWGN and Rayleigh channels (mean ± 1 SD, 10 runs).

**Figure 14:**
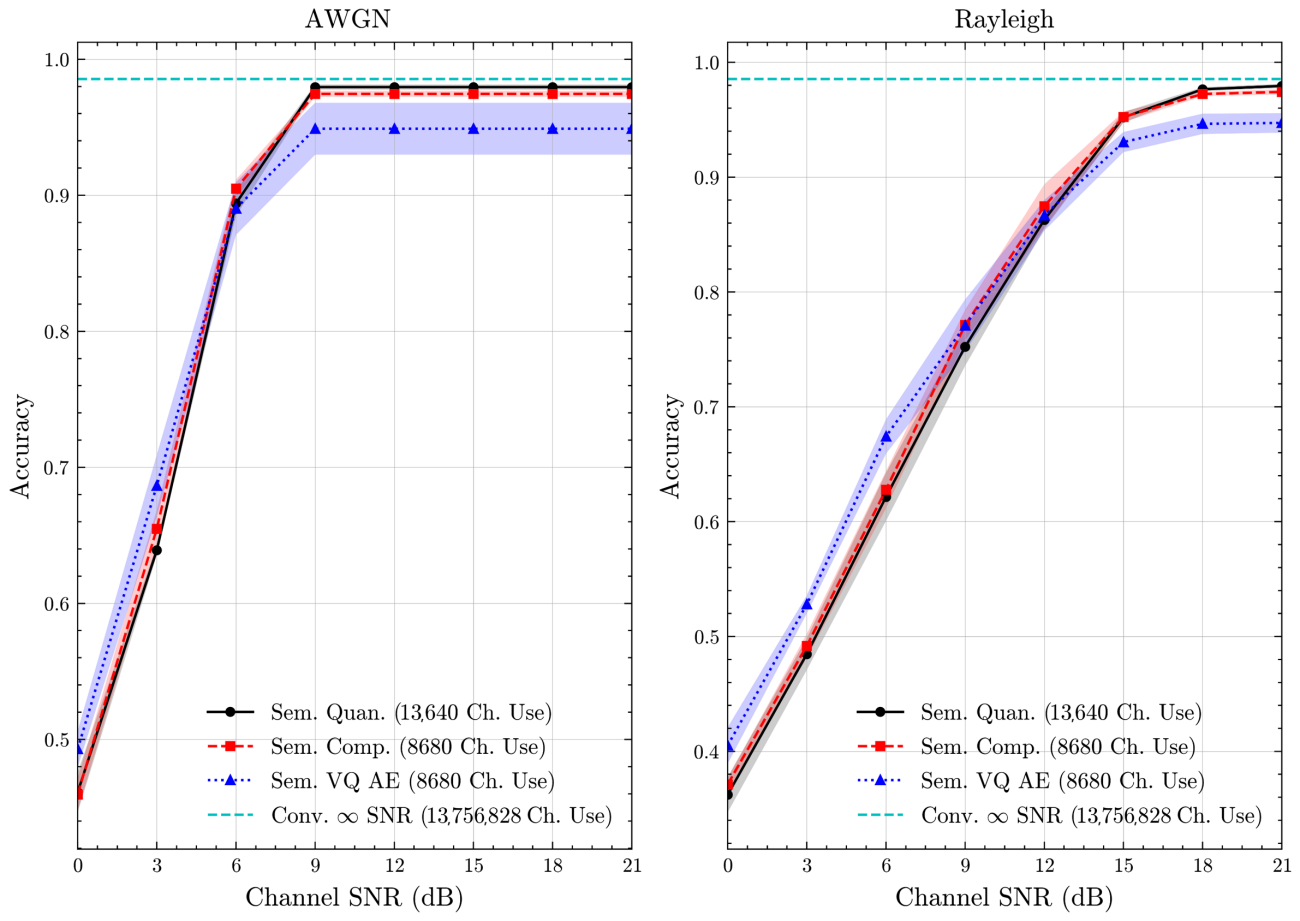
STL 10 dataset classification accuracies over AWGN and Rayleigh channels (mean ± 1 SD, 10 runs).

**Figure 15:**
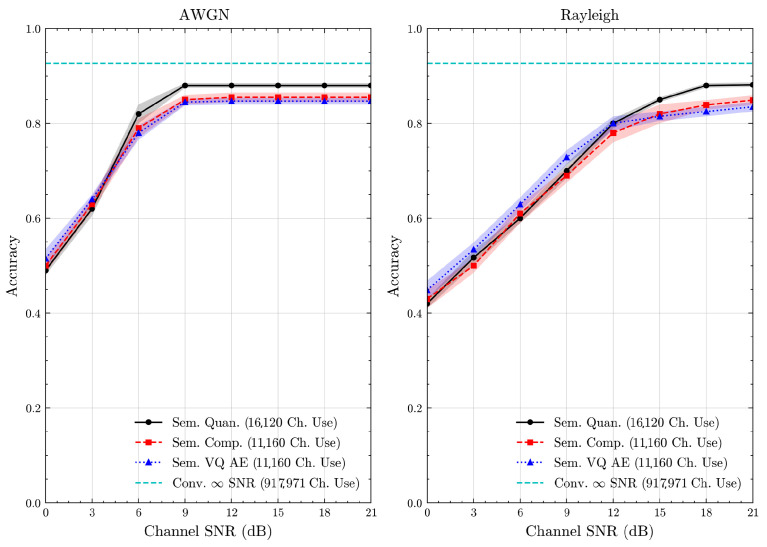
AG’s News dataset classification accuracies with full accuracy range over AWGN and Rayleigh channels (mean ± 1 SD, 10 runs).

**Figure 16:**
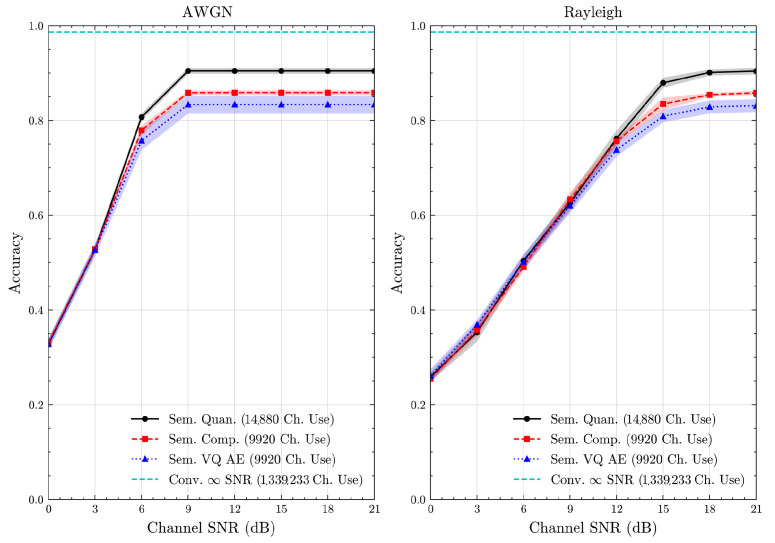
DBPedia 14 dataset classification accuracies with full accuracy range over AWGN and Rayleigh channels (mean ± 1 SD, 10 runs).

**Figure 17:**
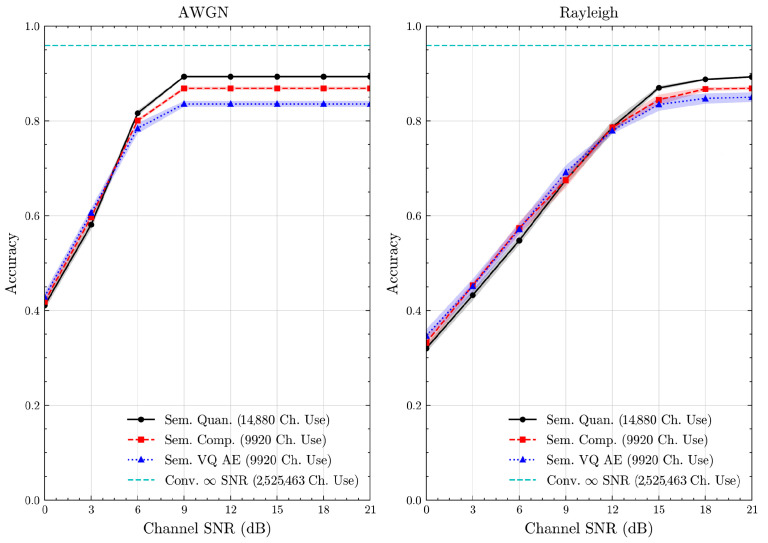
CIFAR 10 dataset classification accuracies with full accuracy range over AWGN and Rayleigh channels (mean ± 1 SD, 10 runs).

**Figure 18:**
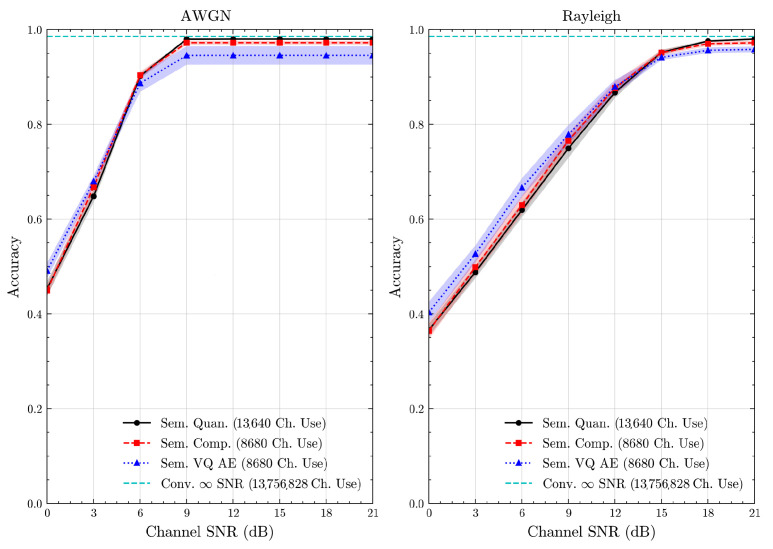
STL 10 dataset classification accuracies with full accuracy range over AWGN and Rayleigh channels (mean ± 1 SD, 10 runs).

**Figure 19:**
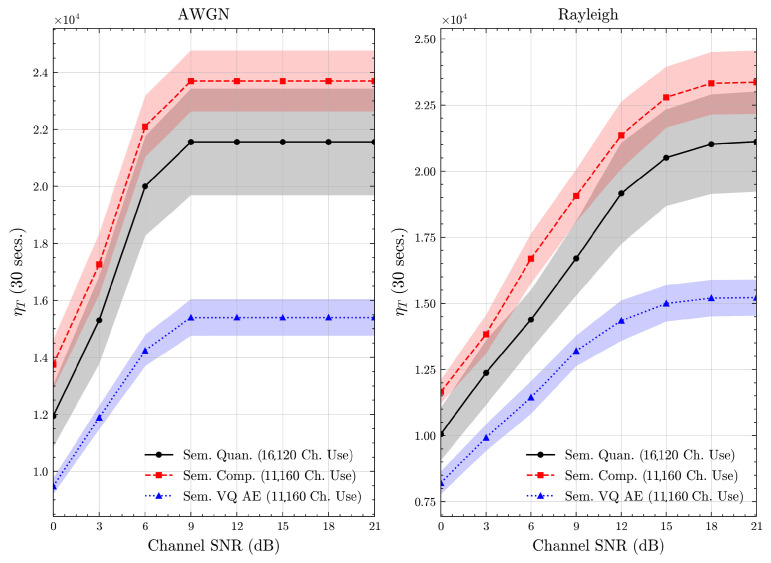
AG’s News dataset system time efficiency scores over AWGN and Rayleigh channels (mean ± 1 SD, 10 runs).

**Figure 20:**
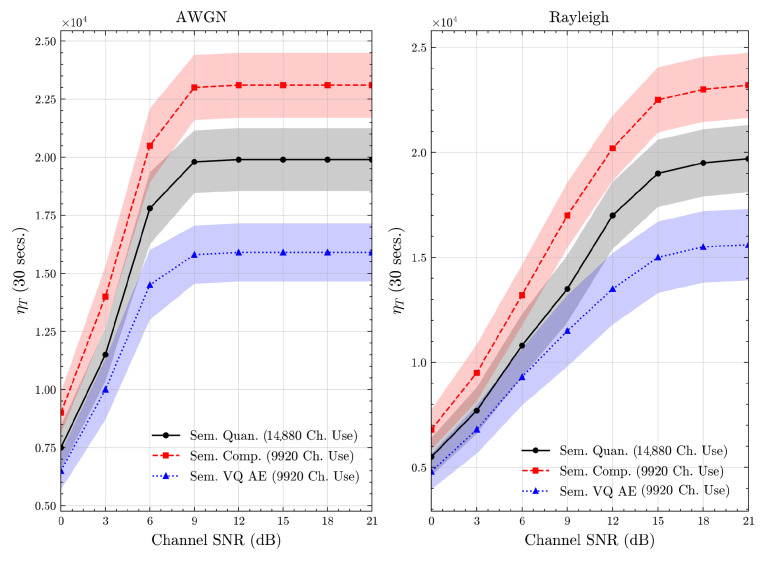
DBPedia 14 dataset system time efficiency scores over AWGN and Rayleigh channels (mean ± 1 SD, 10 runs).

**Figure 21:**
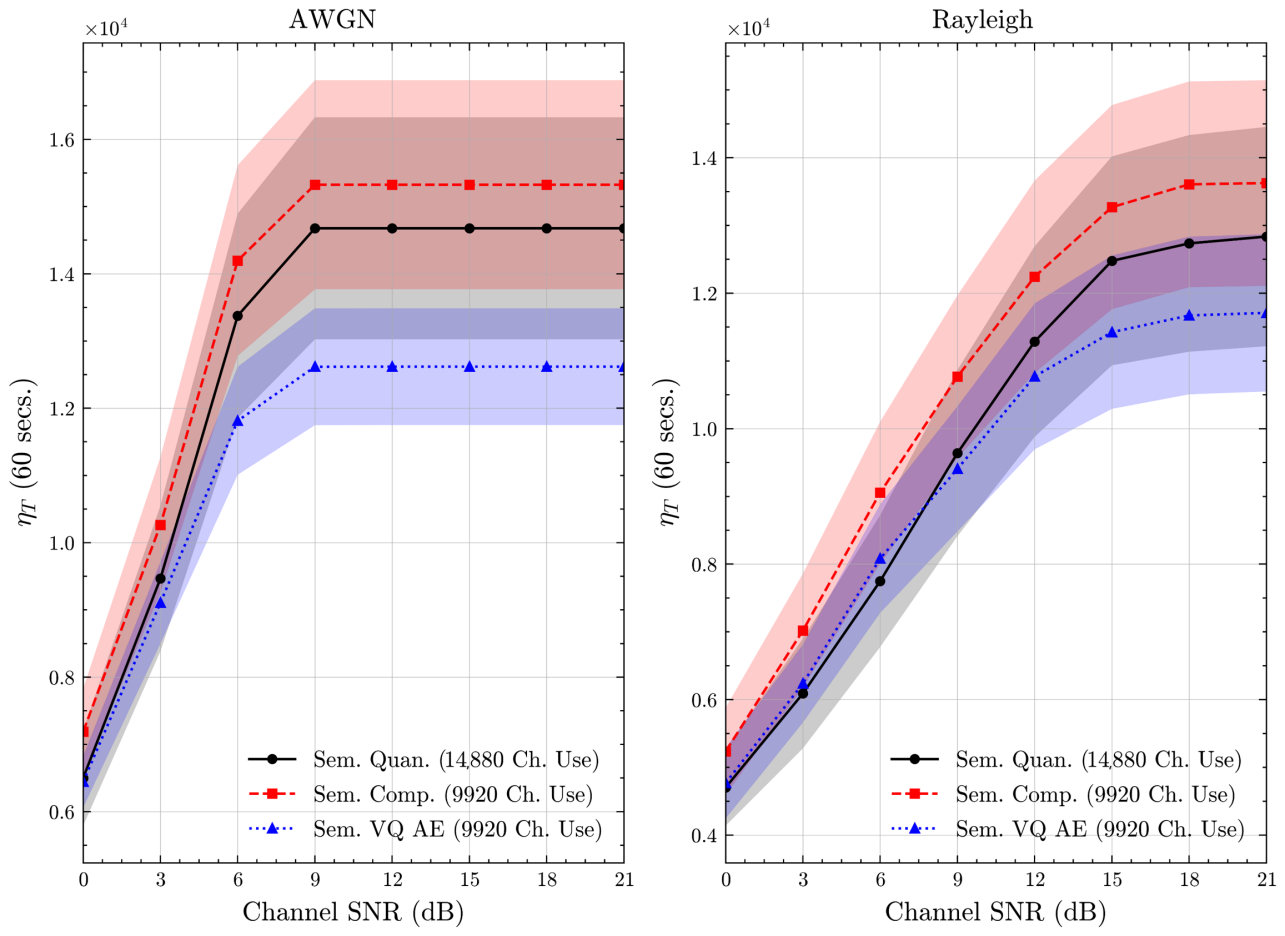
CIFAR 10 dataset system time efficiency scores over AWGN and Rayleigh channels (mean ± 1 SD, 10 runs).

**Figure 22:**
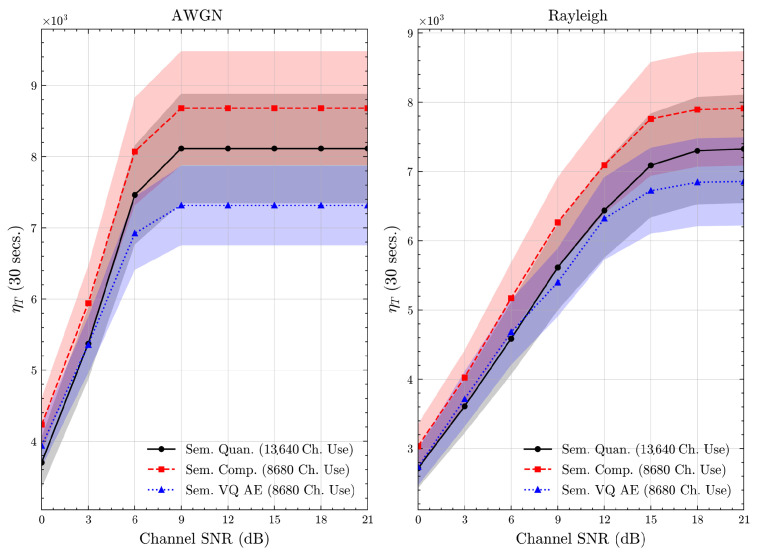
STL 10 dataset system time efficiency scores over AWGN and Rayleigh channels (mean ± 1 SD, 10 runs).

We present the accuracy, time efficiency scores, and corresponding channel uses of the methods for each dataset in Figure 11, Figure 12, Figure 13 and Figure 14 and Figure 19, Figure 20, Figure 21 and Figure 22. Figure 15, Figure 16, Figure 17 and Figure 18 further present these accuracies with the full accuracy range [0,1], as requested by the anonymous reviewer. The performance over wireless channels is consistent with the source compression results in Table 3, as expected. Notably, we highlight the small *n* value, representing the scarce data scenario. For larger *n* values, the methods are expected to converge more closely to the upper limit. Additionally, the variations in convergence across datasets are linked to the semantic structure of the data in the semantic space, which is illustrated for AG’s News in Figure 5.

We allocate time budgets based on modalities, message lengths, and training sizes. For example, CIFAR 10 receives more time than STL 10 due to its larger *n*. Results demonstrate that semantic compression consistently achieves the highest time efficiency, while semantic quantization and semantic VQ AE exhibit inferior performance. We observe that the pre-communication phase of semantic VQ AE, i.e., $t_{train}$, is significantly longer than that of quantization. However, the smaller codebook of VQ AE helps mitigate this during communication, enabling larger *u* values compared to quantization.

We compare the time efficiency of quantization and compression, focusing on the impact of clustering. Compression involves a clustering step that reduces codebook size. This results in an overhead for clustering but leads to a smaller model and faster training for the codeword NN. During communication, quantization’s larger codebook increases computational demands, i.e., the calculation of Equation (4), while compression benefits from a smaller codebook. According to Equation (17), although compression has a similar $t_{train}$, its larger *u* enables it to achieve better efficiency scores than quantization.

We observe that integrating model-based insights with pre-trained models simplifies the objective, e.g., aligning codeword Hamming distances with Euclidean distances in the embedding space. In contrast, purely end-to-end approaches, such as semantic VQ-AE, optimize without any structural guidance, making the process more challenging. This highlights the need to combine model-based reasoning with learning-based methods for effective ML-aided communication. Overall, leveraging semantic redundancies reduces computational load and improves the efficiency of wireless resource utilization. We validate performance via 5 different datasets. Proposed semantic methods are compatible with any pre-trained embedding model that shares semantic relation properties, with semantic quantization and compression methods being particularly adaptable due to their lower computational demands. Proposed methods also allow for the change of the classification block with one suited for other tasks using reconstructed embeddings, without requiring changes to their design.

## 6. Conclusions

In this paper, we have considered classification-oriented multimodal semantic communications. We have used a pre-trained transformer model to extract semantic information, and proposed three different methods for generating their codebooks to minimize semantic distortion. The semantic quantization and compression methods have directly used the available dataset as a codebook, whereas the learning-based method has been trained on the same dataset. We have observed that the proposed methods provide excellent classification accuracy performance, and that pre-training-based methods provide effective compression, resulting in much better system time efficiency with comparable accuracy to their end-to-end learning counterparts. Our study points to the inherent power of utilizing pre-trained models at the edge and demonstrates that with an effective “fine tuning” of codewords, we can have efficient semantic communications.

Future work along these lines include communicating multimodal semantics in multi-node task-oriented networks with personalized tasks.

## Figures and Tables

**Table 1:** Summary of AG’s News, DBPedia 14, CIFAR 10, and STL 10.

Dataset	Modality	Classes	Train Size
AG’s News	Text	4	120,000
DBPedia 14	Text	14	560,000
CIFAR 10	Image	10	50,000
STL 10 *	Image	10	11,000

* The dataset consists of 5000 labeled training and 8000 labeled test samples. Given the limited number of samples, we merge the train and test sets, then extract 2000 samples for the test set, leaving 11,000 samples for training.

**Table 2:** Simulation settings.

Parameter		AG’s News	DBPedia 14	CIFAR 10	STL 10
*n*		5000	3000		1500
Class. Block	Batch Size	128			
	$n_{\mathrm{epochs}}$	15			
	LR Sched.	Exponential LR			
	$\gamma_{\mathrm{scheduler}}$	0.75			
	Initial LR	0.001			
	Optimizer	$AdamW(\beta_{1}=0.9,\beta_{2}=0.999,w_{\mathrm{decay}}=0.01)$			
Codeword NN	Batch Size	128			
	$n_{\mathrm{epochs}}$	Semantic Quantization: 6 Semantic Compression: 10			
	LR Sched.	Exponential LR			
	$\gamma_{\mathrm{scheduler}}$	0.97			
	Initial LR	0.001			
	Optimizer	$AdamW(\beta_{1}=0.9,\beta_{2}=0.999,w_{\mathrm{decay}}=0.01)$			
	ϵ	1			
	$\left(\alpha_{1},\alpha_{2},\alpha_{3}\right)$	(0.25, 0.5, 0.25)			
Semantic VQ AE	*k*	64			
	α	4			
	Batch Size	128			
	$n_{\mathrm{epochs}}$	25			
	LR Sched.	Exponential LR			
	$\gamma_{\mathrm{scheduler}}$	0.9	0.98		
	Initial LR	0.005			
	Optimizer	$AdamW(\beta_{1}=0.9,\beta_{2}=0.999)$			
	$w_{\mathrm{decay}}$	0.0001		0.00025	0.0005
	SNR_min_	0 (dB)			
	SNR_max_	21 (dB)			

**Table 4:** Example of assigned messages using AG’s News dataset.

Method	Corresponding Message
*s*	Wiltshire Police warns about “phishing” after its fraud squad chief was targeted.
$s_{i}$	Do-it-yourself phishing kits are freely available on the Internet, a security firm said Thursday, and they will lead to more scams sent to online consumers.
$s_{l}$	Do-it-yourself phishing kits are being made available for download free of charge from the interest, security watchers have warned.

## Data Availability

The raw data supporting the conclusions of this article will be made available by the authors on request.
